# Risk factors associated with short-term complications in mandibular fractures: the MANTRA study—a Maxillofacial Trainee Research Collaborative (MTReC)

**DOI:** 10.1007/s10006-022-01096-5

**Published:** 2022-07-05

**Authors:** S. Kent, A. Adatia, P. James, K. Bains, A. Henry, C. Blore, B. Dawoud, D. Kumar, C. Jefferies, P. Kyzas, MTReC Collaborators:, D. Sonigra, E. Botha, S. Ooi, M. Bosov, E. Fish, Y. Lin, B. Aslam-Pervez, R. Fletcher, F. Wright, H. Khan, T. Collins, R. Loke, L. Niraj, G. Dhanjal, A. Ghosh, V. Kaneria, C. McIntosh, M. Moksud, C. O Higgins, A. Taha, A. Thompson, G. Tow, J. Wege, F. Sidat, M. Sthankiya, D. Hughes, S. Ng, H. Patel, D. Smyth, C. Craddock, J. Douglas, C. Gordon, S. Iyer, C. Jefferies, P. Sexton, R. Taylor, E. Walshaw, C. Man, J. Sankey, H. Wilcock, A. Nijamudeen, O. Tabbenor, A. Davies, T. Henderson, N. Pigadas, R. Rupchandani, D. Zakai, Y. Coll, B. Dunphy, E. Gruber, Y. Ko, R. Kulkarni, R. Paul, K. Jetty, R. Exley, R. Pancholi, N. Horisk, A. Korobczuk, C. Chandran, A. Dalal, R. Shivam, N. Allison, G. Stonier, F. Dylgjeri, J. Rooney, T. Svoboda, A. Ahmed, S. Farooq, N. Turton, S. Clyde, M. Ritchie, S. Brandsma, H. Nazir, Y. Mousa, S. Choudhury, K. Crawley, E. Offen, A. Iqbal, G. Baniulyte, A. Pamma, O. Yaqoob, D. Britton, C. Sanapala, I. Hashem, S. Icel, A. Goodall, M. Uddin, M. Uddin, A. Aziz, C. Docherty, H. Huguet, M. Kelly, W. Thorley, J. Brar, A. Min, T. Pepper, R. Carr, E. Fahy, A. Geddes, M. Hennigan, C. Simpson, M. Cobb, K. Denholm, J. Neilson, A. Swansbury, A. Dickason, R. Scott, E. Wotherspoon, D. Johnston, D. Murphy, M. Alreefi, N. Althawadi, R. Howells, S. Miles, A. Saadya, J. Sawali, A. Suleiman, S. Olujide, A. Hannah

**Affiliations:** 1 University Hospital of Wales, Cardiff, UK; 2 Aintree University Hospital, Liverpool, UK; 3 Swansea Bay University Health Board, Swansea, UK; 4 University Hospitals of North Midlands NHS Trust, Stafford, UK; 5 North Manchester General Hospital, Manchester , UK; 6 Liverpool Medical School, Liverpool, UK; 7 Leeds Teaching Hospitals NHS Trust, Leeds, UK; 8 Royal Blackburn Teaching Hospital, East Lancashire Hospitals NHS Trust, Blackburn, UK; 9 Bradford Royal Infirmary, Bradford, UK; 10 Bristol Royal Infirmary, Bristol, UK; 11 Derriford Hospital, Plymouth, UK; 12 Forth Valley Hospital, Stirling, UK; 13 Glan Clwyd Hospital, Rhyl, UK; 14 Gloucester Royal Hospital, Gloucester, UK; 15 Hull Royal Infirmary, Hull, UK; 16 James Cook University Hospital, Middlesborough, UK; 17 Kings College Hospital, London, UK; 18 Luton and Dunstable Hospital, London, UK; 19 Manchester Royal Infirmary, Manchester, UK; 20 Newcross Hospital, Wolverhampton, UK; 21 NHS Ayrshire and Arron, Ayrshire, UK; 22 Ninewells Hospital, Dundee, UK; 23 Northampton General Hospital, Northampton, UK; 24 Northwest Anglia Trust, Peterborough, UK; 25 Northwick Park Hospital, London, UK; 26 Pinderfields General Hospital, Wakefield, UK; 27 Poole Hospital NHS Trust, Poole, UK; 28 Prince Charles Hospital, Merthr Tydfil, UK; 29 Princess Alexandria Hospital, Harlow, UK; 30 Queen Alexandra Hospital, Portsmouth, UK; 31 Queen Elizabeth Hospital, Birmingham, UK; 32 Queen Elizabeth University Hospital, Glasgow, UK; 33 Queen’s Hospital, Romford, UK; 34 Queen’s Medical Center, Nottingham, UK; 35 Rotherham General Hospital, Rotherham, UK; 36 Royal Devon & Exeter Hospital, Exeter, UK; 37 Royal Free London NHS Foundation Trust, London, UK; 38 Royal Gwent Hospital, Newport, UK; 39 Royal London Hospital, London, UK; 40 Royal Preston Hospital, Preston, UK; 41 Royal Stoke University Hospital, Stoke, UK; 42 Royal Victoria Infirmary, Belfast, UK; 43 Sheffield Teaching Hospitals, Sheffield, UK; 44 St George’s Hospital, London, UK; 45 St James’s Hospital, Dublin, UK; 46 St John’s Hospital, Livingston, UK; 47 Sunderland Royal Hospital, Sunderland, UK; 48 Torbay Hospital, Torquay, UK; 49 Ulster Hospital, Dundonald, UK; 50 University College Hospital, London, UK; 51 Aberdeen Royal Infirmary, Aberdeen, UK

**Keywords:** Trauma, Mandible, Complications, Risk factors, Infection, Audit

## Abstract

**Introduction:**

Complications following mandibular fractures occur in 9–23% of patients. Identifying those at risk is key to prevention. Previous studies highlighted smoking, age and time from injury to presentation as risk factors but rarely recorded other possible confounders. In this paper, we use a collaborative snapshot audit to document novel risk factors and confirm established risks for complications following the treatment of mandibular fractures.

**Methods:**

The audit was carried out by 122 OMFS trainees across the UK and Ireland (49 centres) over 6 months, coordinated by the Maxillofacial Surgery Trainees Research Collaborative. Variables recorded included basic demography, medical and social history, injury mechanism and type, management and 30-day outcome.

**Results:**

Nine hundred and forty-seven (947) patients with fractured mandibles were recorded. Surgical management was carried out in 76.3%. Complications at 30 days occurred 65 (9%) of those who were managed surgically. Risk factors for complications included male sex, increasing age, any medical history, increasing number of cigarettes smoked per week, increasing alcohol use per week, worse oral hygiene and increased time from injury to presentation.

**Discussion:**

We have used a large prospective snapshot audit to confirm established risk factors and identify novel risk factors. We demonstrate that time from injury to presentation is confounded by other indicators of poor health behaviour. These results are important in designing trial protocols for management of mandibular fractures and in targeting health interventions to patients at highest risk of complications.

## Introduction

Mandibular fracture is the second most common facial fracture and represent a significant proportion of patients presenting to OMFS [1]. Complications following mandibular fractures may result in significant morbidity and occur in 9.8 to 23.7% of patients [2]. Complications can be broadly divided into those requiring further surgery, those requiring administration of a medication, and those which do not require further action [3]. Understanding patient and treatment factors associated with complications is the key to reducing complication rates.

The only patient characteristic conclusively shown to be associated with complications is smoking [4–7]. Other possible patient factors include increasing age [5], depression [8] and ‘poor patient compliance’ [8]. In previous studies, smoking has been treated as a binary variable with no study identifying a dose-dependant effect of number of cigarettes on complications rates. Sex, alcohol use, oral hygiene and past medical history have never previously been shown to be risk factors for complications following management of mandibular fracture.

Injury and treatment characteristics associated with complications may include increased time from injury to surgical fixation [9] and increased time from injury to initiation of antibiotic prophylaxis [10]. However, a contemporary study has suggested that the use of any preoperative antibiotics may be associated with increased complication rates [11] and a recent meta-analysis shows no difference in complications rates with various antibiotic regimens [2]. The use of semi elective fixation in a growing cohort suggests that time from injury to fixation is not always regarded as a risk factor for complication [13]. Further studies are required to establish which of the many treatment characteristics are associated with complications in mandibular fractures.

**Fig. 1:**
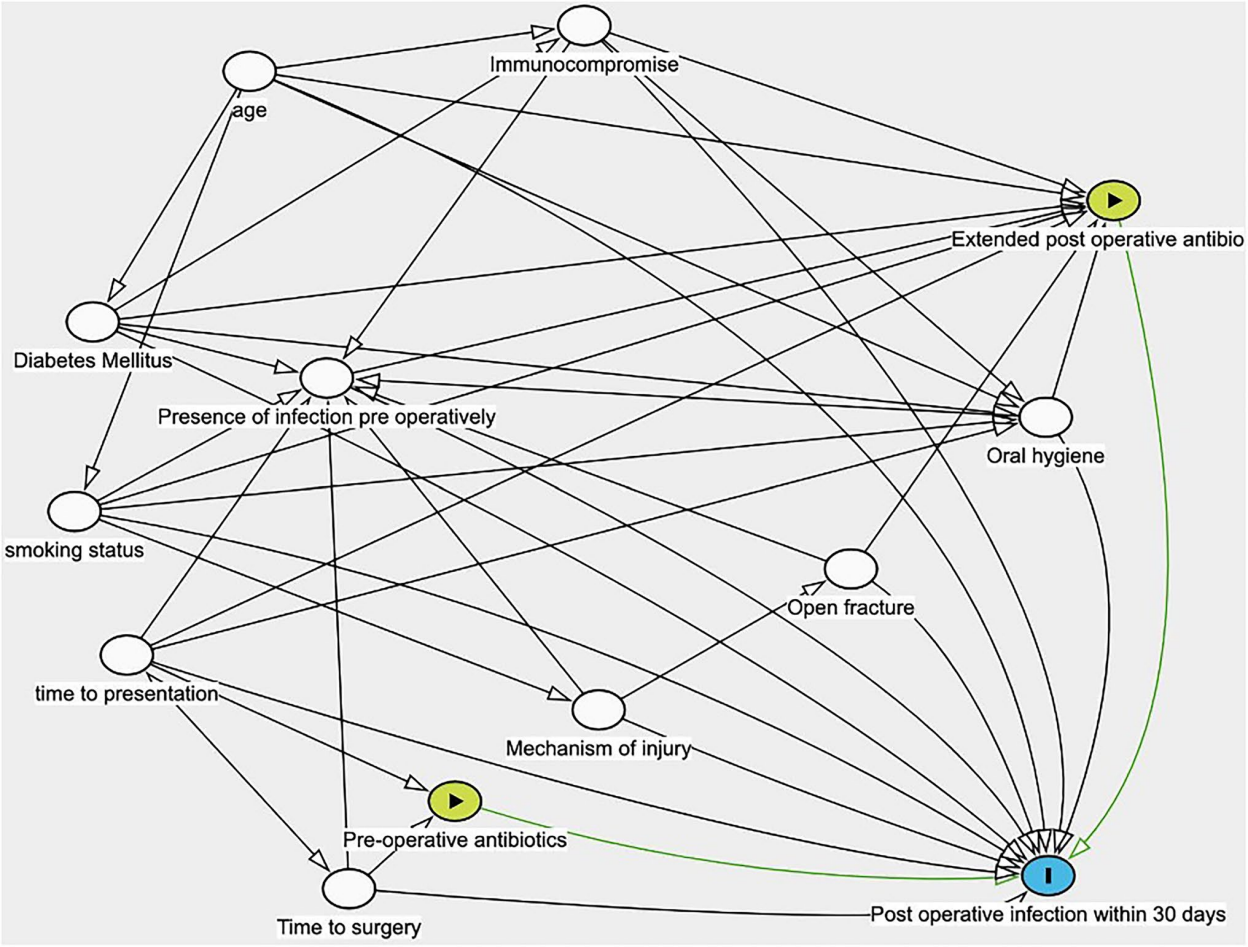
Direct acyclical graph showing potential risk factors for complications in mandibular fractures and interaction between these risk factors

Because there are so many potential factors associated with complications (Fig. 1), large numbers of cases are required to give sufficient statistical power and allow subgroup analysis. To gather adequate data, a collaborative approach is required. We have used a prospective, trainee-led snapshot audit to identify risk factors associated with postoperative mandibular fracture complication rates.

## Methods

One hundred and twenty two trainees in Oral and Maxillofacial Surgery (OMFS) across 49 centres in the UK and Ireland have carried out a prospective snapshot audit, between 3 December 2020 and 3 June 2021. The work was coordinated by The Maxillofacial Surgery Trainees Collaborative (MTReC) and was registered as a national audit at each centre. Data recorded in the snapshot audit was routinely gathered as part of usual patient care, and no additional measures or observations were made. Additional ethical approvals were not required.

Data was anonymised at the point of collection and was recorded using the Research Electronic Database Capture (REDCap) database system [14, 15]. REDCap allows collaborators to review and edit data they have collected.

Data gathered by the snapshot audit included patient age, sex, whether the patient was medically fit, smoking status and number of cigarettes smoked per week, alcohol consumption per week, oral hygiene status, mechanism of injury, anatomical position of fracture within the mandible, presence of other injuries, time from injury to presentation and time from injury to surgery, use of post-operative antibiotic prophylaxis, extended course of post-operative antibiotics and 30-day follow-up administration of any courses of unplanned antibiotics or readmission and reason.

Complications were defined as any unplanned administration of antibiotics or readmission to hospital within 30 days of first presentation. Patients with complications were compared to those without complications. Comparison of sex, age, mechanism of injury, smoking status, number of cigarettes smoked per week, alcohol consumption, oral hygiene, American Society of Anaesthesiologists scale (ASA) [16], whether patient had any past medical history, use of antibiotic prophylaxis, position of fracture, time from injury to presentation, time from injury to initiation of antibiotic prophylaxis and time from injury to surgery was made.

Distribution of continuous variables was analysed using histograms, and all variables were found to have nonparametric distribution. Statistical tests used were Kruskal Wallis *H* test for continuous variables, Pearson’s chi-square for binary variables and Kendall’s tau for ordinal variables. Significance level was set at *p* < 0.05.

## Results

Data was collected on 947 patients with fractured mandibles, 759 male and 188 female (4:1). Surgical management was carried out in 722 (76.7%), whilst 225 (23.7%) were managed without surgery. Those managed without surgery included mainly isolated condylar fractures (52.5%), isolated angle fractures (16%) or isolated coronoid or ramus fractures (11.3%). The remaining fractures were a mixture of fracture combinations.

Complications at 30 days occurred in 68 (7.1%) patients; 65 of these were in surgically-managed patients (9%). Complications included unplanned courses of oral antibiotics in 55 patients (54 (7.5%) operatively managed, (1 (0.4%) non-surgically managed), and readmission in 24 patients (22 (3%) surgically managed), 2 (0.9%) non-surgically managed). Readmission was for intravenous antibiotics only (7, 0.7%), revision of ORIF (12, 1.2%), removal and replacement of infected hardware (4, 0.4%), removal of retained tooth (3, 0.3%), removal of infected hardware without replacement (1, 0.1%) and other (7, 0.7%). Some readmitted patients received more than one treatment.

The group with complications at 30 days was compared to the group with no complications, initially in the entire patient group (*n* = 947) then in the surgically managed group (*n* = 722). Factors associated with complications included increasing age, male sex, current smoker status, increasing number of cigarettes smoked per week, increasing number of units of alcohol consumed per week, not being ‘medically fit’, worse oral hygiene, increasing time from injury to presentation and increasing time from injury to initiation of antibiotic prophylaxis. No significant differences were seen between groups for ASA, use of preoperative antibiotic prophylaxis, use of postoperative intravenous antibiotics, use of extended post-operative prophylaxis, time from injury to surgery and time from presentation to surgery (Tables 1 and 2).

**Table 1:** All patients (*n* = 947), divided by presence of unplanned interventions within 30 days

		Unplanned intervention (antibiotics or readmission) (*n* = 68)	No unplanned intervention (*n* = 879)	Kendall’s tau-b OR chi-square	Kruskal–Wallis H	df	*p*
Age (median, range)		35, 16–72	31, 3–94		4.482	1	.034
Sex (%)	Male	48 (70.6)	711 (80.9)				
	Female	20 (29.4)	168 (19.1)	4.208		1	.033
Smoking status (%)	Non-smoker	13 (4.3)	292 (95.7)				
	Ex-smoker	5 (11.1)	40 (88.9%)				
	Smoker	46 (9.7)	427 (90.3)	− 0.102			< 0.001
Cigarettes smoked	1–5/day	3 (4.2)	68 (95.8)				
	6–10/day	12 (8.8)	125 (91.2				
	11–20/day	10 (14.8)	115 (85.2)				
	20 + /day	8 (11.4)	62 (88.6)	0.91			.004
Alcohol unit numeric (%)	Teetotal	5 (3)	163 (97)				
	< 14 units/week	19 (5.2)	346 (94.8)				
	> 15 units/week	19 (10.9)	156 (89.1)				
	Alcohol dependant	14 (18.2)	63 (81.1)	0.149			< 0.001
ASA numeric (%)	1	15 (4.8)	298 (95.2)				
	2	49 (8.6)	518 (91.4)				
	3	3 (4.8)	59 (95.2)				
	4	1 (25)	3 (75)	0.52			0.069
Significant past medical history	Yes	48 (8.9)	491 (91.1)				
	No	20 (4.9)	388 (95.1)	5.584		1	0.012
Dentate		67 (98.5)	850 (96.7)	0.627		1	0.369
Oral hygiene numeric	Good	8 (3.8)	201 (96.2)				
	Fair	30 (6.6)	428 (93.4)				
	Poor	29 (11.8)	217 (88.2)	0.104			0.001
Position of fracture (Brown classification ^26^) (%)	1	6 (4.8)	120 (95.2)				
	2	14 (20.6)	191 (93.2)				
	2c	1 (4.5)	21 (95.5)				
	3	11 (10)	99 (90)				
	3c	18 (11.5)	138 (88.5)				
	4	15 (6.1)	230 (93.9)				
	4c	0	23 (100)				
	5	3 (5)	57 (95)	− 0.002			0.924

**Table 2:** Surgically managed patients (*n* = 722), divided by presence of unplanned interventions within 30 days

		Unplanned intervention (antibiotics or readmission) (*n* = 65)	No unplanned intervention (*n* = 652)	Kendall’s tau-b OR chi-square	Kruskal–Wallis H	df	*p*
Age (median, range)		36 (16.72)	29 (3.94)		8.331	1	.004
Sex (%)	Male	46 (7.7)	550 (92.3)				
	Female	19 (15.7)	102 (84.3)	− 0.104			.023
Smoking status (%)	Non-smoker	12 (5.9)	191 (94.1)				
	Ex-smoker	5 (14.7)	29 (85.3)				
	Smoker	44 (11.2)	350 (88.8)	− 0.087			0.008
Cigarettes smoked	1–5/day	3 (4.8)	59 (95.2)				
	6–10/day	11 (9.6)	103 (90.4)				
	11–20/day	19 (16.5)	96 (83.5)				
	20 + /day	8 (15.4)	44 (84.6)	0.117			0.013
Alcohol units numeric (%)	Teetotal	5 (4.6)	103 (95.4)				
	< 14 units/week	18 (6.3)	266 (93.7)				
	> 15 units/week	18 (12.7)	124 (87.3)				
	Alcohol dependant	14 (23.3)	46 (76.7)	0.157			< 0.001
ASA numeric (%)	1	14 (6.3)	207 (93.7)				
	2	47 (10.5)	401 (89.5)				
	3	3 (6.8)	41 (93.2)				
	4	1 (33.3)	2 (66.7)	0.53			0.112
Medically fit?	Yes	19 (6.1)	290 (93.9				
	No	46 (11.3)	362 (88.7)		5.606	1	0.018
Oral hygiene numeric	Good	8 (5.5)	137 (94.5)				
	Fair	27 (7.5)	335 (92.5)				
	Poor	29 (14.8)	167 (85.2)	0.112			0.003
Pre-operative antibiotic use? (%)	Yes	58 (89.2)	581 (89.1)				
	No	7 (10.7)	67 (10.2)		0.012	1	0.833
Post-operative antibiotic use? (%)	Yes	60 (92.3)	601 (92.3)				
	No	5 (7.7)	50 (7.7)		0.000	1	1.00
Extended course of post-operative antibiotics? (%)	Yes	47 (72.3)	446 (68.4)				
	No	18 (27.7)	205 (31.4)		0.397	1	0.577
Estimated time of injury to antibiotics, hours minutes median, (IQR)		13 h (34 h)	7 h (23 h)		5.131	1	0.023
Time from injury to presentation, hours, minutes median (IQR)		13 h 20 m, (34 h 7 m)	6 h 59 m (22 h 59 m)		5.111	1	0.024
Time from injury to surgery hours, minutes median (IQR)		46 h 10 m (48 h 29 m)	50 h (81 h 57 m)		.662	1	0.416

Further analysis was carried out to determine how time from injury to presentation varied with other indicators of health behaviour. Significantly longer times from injury to presentation were seen in patients with other indicators of poor health behaviours including smoking status, number of cigarettes smoked per week, number of units of alcohol consumed per week and worse oral hygiene (Table 3).

**Table 3:** Surgically managed patients (*n* = 722), time from injury to admission

Test statistics		Time from injury to presentation (hours, minutes) (median, IQR)	Kruskal–Wallis H	df	*p*
Age					
Sex	Male (*n* = 596)	8 h (23 h 57 m)			
	Female (*n* = 121)	6 h (21 h 54 m)	1.581	1	0.209
Smoking status	Non-smoker (*n* = 203)	5 h 2 m (13 h 31 m)			
	Ex-smoker (*n* = 34)	12 h 29 m (32 h 35 m)			
	Smoker (*n* = 394)	11 h 43 m (36 h 59 m)	24.497	2	0.000
Cigarettes smoked	1–5/day (*n* = 62)	6 h (36 h 5 m)			
	6–10/day (*n* = 114)	12 h 15 m (30 h 59 m)			
	11–20/day (*n* = 115)	17 h (58 h 27 m)			
	20 + /day (*n* = 52)	19 h 39 m (57 h 10 m)	8.969	3	0.030
Alcohol units numeric	Teetotal (*n* = 108)	5 h 53 m (22 h 52 m)			
	< 14 units/week (*n* = 284)	8 h (22 h 48 m)			
	> 15 units/week (*n* = 142)	11 h 15 m (35 h 36 m)			
	Alcohol dependant (*n* = 60)	14 h 39 m (60 h 22 m)	13.519	3	0.004
Oral hygiene numeric	Good (*n* = 145)	4 h 59 m (22 h 35 m)			
	Fair (*n* = 362)	7 h 35 m (22 h 35 m)	25.737	2	0.000
	Poor (*n* = 196)	13 h 59 m (44 h 41 m)			

## Discussion

We have used a large prospective snapshot audit to identify novel risk factors and confirm established risk factors for complications following mandibular fracture. For the first time, we have identified a dose-dependant effect of alcohol and smoking on complications and identified that time from injury to presentation is associated with other poor health behaviours.

Previous reviews of complications in mandibular fractures have mainly used a retrospective design to assess risk factors in cohorts of between 75 and 409 participants but are inadequate to conclusively establish risk factors for complications [2]. A previously published 2017 paper had a large number of participants (*n* = 642) but only looked at antibiotic use and timing rather than patient related factors [10]. The largest retrospective study of risk factors (*n* = 953) for complications in mandibular fractures did not address patient factors such as alcohol use, past medical history or timing of presentation and surgery or antibiotic use [5].

Smoking is a well-established risk factor for surgical complications. A large-scale prospective study noted 14.9% of smokers to have complications including infection and malunion in comparison with 8.5% of non-smokers [17]. Calcium absorption is slower in smokers [18] and in animal studies simulating the effects of smoking tobacco, collagen synthesis is reduced which may lead to instability at the fracture fixation site [19]. The increased risk of surgical site infection in those who smoke may be due to the hypoxic and vasoconstrictive effects of nicotine and carbon monoxide [18]. Our study is the first to show a dose-dependant effect of cigarette smoking with more complications in heavier smokers.

There were also more complications in patients who consumed more units of alcohol per week. The rate of complications was the highest in those who were alcohol dependent (23.3%). A previous study noted a similar complication rate of 17.1% in those who were alcohol abusers [17]. The chronic use of alcohol is known to alter bone metabolism with features such as decreased vitamin D metabolites, hypocalcaemia and hypercalciuria [20]. It is also associated with T-cell suppression which can lead to greater host susceptibility to bacterial colonisation and surgical site infection [21].

Two previous studies have identified past medical history as a predictor of complications [7, 8]. In one study, depression was significantly associated with complications, though the sample size was small (317) and failed to show smoking as a risk factor [8]. Here, we showed that increasing ASA category was not associated with complications, though small numbers with high ASA scores make this result unreliable. Many of the novel and established risk factors for complications following mandibular fracture (smoking, alcohol, oral hygiene, failure to comply with post-op instructions [8, 7], depressive illness [8], male sex [22]) are also indicators of poor health behaviours.

Both time from injury to fixation and time from injury to initiation of antibiotic prophylaxis have been identified as possible risk factors for complications in previous studies [9, 10]. Our results show that time from injury to fixation was not associated with complications at 30 days (median 48 h 10 m vs 50 h, *p* = 0.416). This agrees with several other studies [9, 23, 24]. In some of them, there was no significant difference in mandibular fracture whose repairs were delayed over 72 h, or any other time delay. The authors have suggested that the vascular supply of the head and neck region is sufficient to prevent or delay the formation of sequestrum [24].

The current audit shows that increased time from injury to presentation and increased time from injury to initiation of antibiotic prophylaxis were significantly associated with complications at 30 days (time from injury to presentation median 6 h 59 m vs 13 h 20 m, *p* = 0.024). This may be because early administration of antibiotics decreases infection rates, which is the conclusion drawn by a large previous retrospective study ^10^. However, this study did not take account any potential confounding factors.

**Fig. 2:**
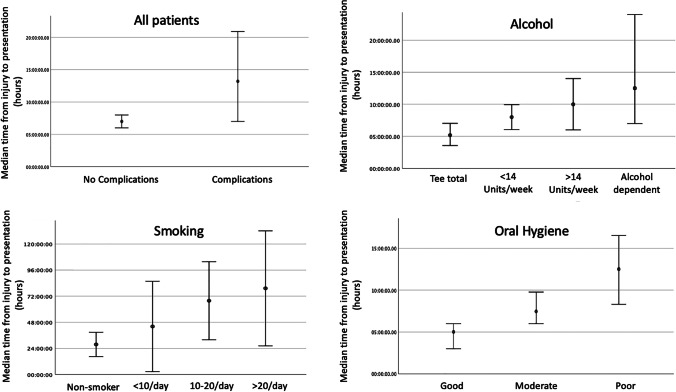
Subgroup analysis of time from injury to presentation with alcohol use, smoking and oral hygiene

Time from injury to presentation (but not time from injury to fixation) may be an example of poor health behaviour. To test this, we measured time from injury to presentation as a dependant variable with indicators of health behaviours as independent variables (Table 3). This has shown, for the first time, that increased time from injury to presentation is associated with smoking, increased alcohol use and worse oral hygiene. These results suggest that time from injury to presentation is confounded by other health behaviours (Fig. 2). It is impossible to establish causation from an observational study and whilst it appears that poor health behaviours (smoking, alcohol, oral hygiene, time to from injury to presentation) are closely associated, further randomised controlled studies are required to establish the role of each.

Antibiotic prescribing practices for post-operative mandibular fractures vary widely [12]. In our snapshot audit, there was no significant difference in complication rates between pre-operative vs no antibiotic prophylaxis, post-operative antibiotics vs no post-operative antibiotics or use of extended post-operative prophylaxis vs no extended prophylaxis. Our group has recently completed a systematic review of the literature relating to antibiotic use in mandibular fractures and concluded that there is no difference in complication rates with different regimens [2]. In 2020, the Surgical Infection Society published guidelines for antibiotic use in patients with traumatic facial fractures, recommending no pre-operative antibiotic prophylaxis for operative mandibular fractures in adults, and that post-operative antibiotics should not be prescribed for more than 24 h [25]. The results from this snapshot audit agree with these guidelines.

Though this is one of the largest prospectively gathered snapshot audits of complications in mandibular fractures, there are several limitations. Data was collected by trainees at 49 centres across the UK; therefore, checks of data quality were carried out remotely. Grading of oral hygiene was subjective. Decayed, missing or filled teeth (DMFT) or plaque and bleeding score indices could have been used but are not recorded routinely in most units. The large number of potential confounding factors complicates further analysis and highlights the need for randomised trials.

Thirty-day follow-up may be too short to record many of the complications which occur following mandibular fractures, and additional factors such as the type of plate, intermaxillary fixation, nutritional status, length of procedure and seniority of operator were not recorded. However, as this snapshot audit was carried out by UK trainees in OMFS who usually change job every year or 6 months, this pragmatic dataset and follow-up period was chosen to ensure consistency and completeness in data collection.

## Conclusions

We have used a large prospective snapshot audit to identify novel risk factors for complications following mandibular fracture (sex, number of cigarettes smoked per week, alcohol use, oral hygiene) and confirm established risk factors (smoking status, age, medical history, time from injury to presentation). For the first time, we have identified a dose dependant effect of alcohol and smoking on complications and identified that time from injury to presentation is associated with other poor health behaviours.

These results are important in design of protocols for managing mandibular fractures and in targeting health interventions to patients at highest risk of complications.
